# Perspectives on Current Attitudes, Enablers, and Barriers to Obtaining Surgical Informed Consent for Doctors-in-Training

**DOI:** 10.7759/cureus.40958

**Published:** 2023-06-26

**Authors:** Mary Teoh, Daniel Jia Wei Lee, David Cooke, Munyaradzi G Nyandoro

**Affiliations:** 1 General and Colorectal Surgery, Sir Charles Gairdner Hospital, Perth, AUS; 2 General Surgery, Fiona Stanley Hospital, Perth, AUS; 3 General and Colorectal Surgery, Fiona Stanley Hospital, Perth, AUS

**Keywords:** barriers, enablers, attitudes, doctors-in-training, surgical informed consent

## Abstract

Background

Surgical informed consent (SIC) is paramount in modern-day litigious surgical practice, yet numerous complaints remain about the consenting process. This paper investigated current attitudes, enablers, and barriers to obtaining SIC in clinical practice for doctors-in-training (DiT).

Methodology

Self-reported SIC practice among DiT (N=1,652) across three metropolitan health service regions in Western Australia (WA) was surveyed using a de-identified 20-item multiple response ranking, dichotomous quantitative and qualitative online survey. Data were analyzed using Statistical Package for the Social Sciences (SPSS) version 27 (IBM Corp., Armonk, NY, USA).

Results

The response rate was 23% (n=380). There was an even distribution of key demographics across all three health regions; the median postgraduate year (PGY) was two. Only 57.4% of DiT strongly felt comfortable and confident obtaining a SIC. Of the responders, 67.4% correctly identified key SIC components. There were significant positive associations between comfort and confidence with obtaining SIC and the seniority level of the DiT (p<0.001), identification of SIC components (p<0.001), and prior training in SIC (p<0.001). Most DiTs highlighted the necessity for formal SIC training with a preference for interactive workshops supported by e-learning modules.

Conclusions

Most DiTs can identify the key factors that constitute a valid SIC; however, the practical conversion of this skill could be better. The key enablers to improved SIC techniques were well-supported departments, with further training and clear guidelines within the institutions. The identified barriers were time constraints, inexperience, and a lack of senior support. Future practices and interventions should address these key barriers while promoting the enablers of sustainable and efficient SIC practice.

## Introduction

Surgical informed consent (SIC) is paramount in modern-day litigious surgical practice. The balance of legal and ethical paradigms makes obtaining informed consent challenging while ensuring appropriate discussion of risks, including material risks. A material risk is one in which, in a particular case, a reasonable person in the patient’s position would likely attach significance [1,2]. The shift in focus from a doctor-centered view (also known as the Bolam principle, a medical ethos founded on trust and the assumption that “doctor knows best”) to the current model of SIC (with a focus on “reasonable patient standard”) was changed in Australia due to several medicolegal cases, the most prominent of which was the Rogers versus Whitaker case in 1992 [1].

The Rogers versus Whitaker case describes Whitaker having a complication from ophthalmic surgery resulting in sympathetic ophthalmia in her unaffected eye, causing her to become blind in both eyes. Despite this risk being 1:14,000, the High Court deemed it a “material risk to the patient,” considering it an essential material risk that her surgeon should have discussed as part of the duty of care. As such, the current position is that the doctor “must warn a patient of a material risk inherent in the proposed treatment” [2]. Despite this precedent set for three decades, the medical indemnity services still report that close to 12% of complaints are related to the consenting process [3].

Key components of the SIC process include the following: (i) description of the disease process, need for surgical intervention, and proposed operation, (ii) discussion of the material risks (particular to the patient) and the anticipated benefits of the operation, (iii) discussion of different available surgical techniques and alternative management options, (iv) acknowledgment of possibilities of uncertainties in the diagnosis and other expected outcomes, (v) disclosure of who performs the operation, and (vi) assessment of the patient’s perspective and understanding (including capacity) [4].

The consent process involves demonstrating the legal responsibilities of performing SIC and completing the relevant documentation and is a crucial standard domain for all medical programs [5]. However, throughout the literature, deficits in clinicians’ familiarity with the medicolegal requirements of SIC have emerged that affect key fundamentals of capacity assessment, adequate disclosure, and legal documentation [6,7]. These deficiencies are reflected in global studies that identified various attitudes and barriers, such as lack of training, junior clinicians having to undertake SIC for unfamiliar procedures, or difficulty engaging the patient in the consenting process [7-10].

Given that education and understanding of the essential components of SIC should be taught in the junior stages of the clinician’s career, we sought to gain insight into the enablers and barriers to the clinicians’ understanding and skills with SIC.

This article was previously presented as a meeting abstract at the 2021 Royal Australasian College of Surgeons Annual Scientific Congress (ASC) on May 11th, 2021, in Melbourne and May 6th, 2019, in Bangkok.

## Materials and methods

Study population

In Australia, the natural progression of the medical career begins as an intern, then a resident, registrar, fellow of a specialty college, and finally, a consultant. An intern is a postgraduate year 1 (PGY1) doctor with provisional registration. A resident is at least PGY2 and possesses general registration. A registrar is usually PGY3 and above and can be accredited by a specialty college, meaning undergoing specialist training. Registrars not enrolled in an advanced specialist training program are referred to as unaccredited or service registrars. Registrars supervise both interns and residents. This paper acknowledges that the role and level of resident and registrar as defined in Australia may differ internationally; therefore, the postgraduate year level associated with these roles may help the readers match their own system.

All 1,652 doctors-in-training (DiT) interns (n=322), residents (n=761), registrars (n=484), and fellows (n=85) from three metropolitan regions in Western Australia (WA) were invited via multimedia formats (email, hyperlinks, and QR codes on societies’ social media sites and at grand rounds and teaching sessions) to participate in an online, de-identified survey. The self-reported SIC practice survey was conducted between September 2018 and March 2019 and contained a mix of dichotomous (yes/no), multiple responses, and free text completion items (Appendices: Supplemental online materials - pipetted survey questionnaire). Multi-platform reminders were sent to improve the response rate. Essential components such as participants’ comfort and confidence levels with obtaining SIC were assessed using a 5-point Likert scale.

Statistical analysis

Baseline characteristics and self-reported practice were described using mean (± standard deviation), median (interquartile range), and frequencies/proportions as appropriate. Outcomes for continuous unpaired variables were analyzed with the nonparametric Mann-Whitney U test. Dichotomous outcomes were compared between groups using χ2 or Fisher’s exact tests with no adjustment for multiple comparisons. For the primary outcome, the relative comfort and confidence in obtaining a SIC was captured using the Likert scale and expressed as a proportion and 95% confidence interval. A secondary analysis of the primary outcome was performed using univariate analysis to assess significant associations. The correlation between two quantitative variables was evaluated using Spearman’s rank correlation test. All analyses were performed using Statistical Package for the Social Sciences (SPSS) version 27 (IBM Corp., Armonk, NY, USA), and a two-tailed p-value of <0.05 was considered statistically significant.

Ethics

This project was approved as a quality improvement project of negligible risk with authority to publish by the lead Human Research Ethics Sub-Committees on Safety, Quality Improvement, and Governance (reference #28025, 28098). All participants provided informed consent for the publication of their de-identified data.

## Results

Demographics

The response rate was 23% (n=380), with equal distribution of key demographics across all three health regions. Most respondents were between postgraduate years 1 and 4, with the majority being residents. Two-thirds of respondents had no prior SIC training. Of those with training, 42.1% received it in medical school. Most respondents agreed or strongly agreed that formal SIC training was necessary. In addition, 67.4% understood the essential components of SIC, with most (85%-92%) correctly identifying separate components of SIC (Table 1).

**Table 1:** Demographic and SIC training and education characteristics of respondent DiT Values are the number of participants (%) unless otherwise indicated. SIC: surgical informed consent, DiT: doctor-in-training

Variable	Proportion	Number
N=380	(%)	(n)
Health service		
South Metropolitan	35.8	136
North Metropolitan	32.9	125
East Metropolitan	31.3	119
Gender		
Male	43.2	164
Female	53.2	202
Undeclared	3.7	14
Position		
Intern	10.8	41
Resident medical officer	50.3	191
Registrar	34.5	131
Fellow	4.5	17
SIC training and education characteristics of respondent DiT		
	(%)	(n)
Received SICtraining		
Yes	66.8	254
No formal training	33.2	126
Level of training when first received instruction on obtaining SIC for procedures		
Medical school	42.1	160
Internship	16.1	61
Residency	7.4	28
Registrar	1.3	5
No formal training	33.2	126
Opinion on the necessity of formal training on obtaining SIC		
Strongly agree	68.7	261
Agree	28.4	108
Neutral	2.9	11
Preferred format for education/resources		
Pocket guides/handbooks	26.8	102
Lecture series	10.5	40
Video/web-based modules	61.6	234
Interactive tutorials/practice	1.1	4

Components of SIC

Most respondents (67.4%) understood what constituted all the essential components of SIC. However, identifying the separate SIC components was variable, ranging between 85% and 96%. The recording of consent received the highest recognition rate at 94.5%, and the recording of the surgeon’s signature was the lowest at 85% (Table 2).

**Table 2:** Understanding of essential SIC components by respondent DiT Values are the number of participants (%) unless otherwise indicated. SIC: surgical informed consent, DiT: doctor-in-training

Variable	Proportion	Number
N=380	(%)	(n)
Age patients can give SIC		
14	3.9	15
16	49.5	188
18	46.6	177
Is the evaluation of competence an essential component of SIC?		
Yes	90	342
No	10	38
Is patient education an essential component of SIC?		
Yes	92.9	353
No	7.1	27
Is recording the consenting process an essential component of SIC?		
Yes	94.5	359
No	5.5	21
Is recording the patient’s signature an essential component of SIC?		
Yes	89.5	340
No	10.5	40
Is recording the surgeon’s signature an essential component of SIC?		
Yes	85.3	324
No	14.7	56
Is recording the date of consent an essential component of SIC?		
Yes	90	342
No	10	38
Are all the above factors essential components of the SIC?		
Yes	67.4	256
No	32.6	124

SIC practice

This survey shows that DiTs have good self-reported practices in obtaining SIC. Most routinely check for competency and discuss key tenants such as diagnosis, indications, risks, benefits, potential complications, and alternative management options. However, only 57.4% of the respondents felt comfortable or confident obtaining SIC (Table 3).

**Table 3:** Current SIC practice of respondent DiT Values are the number of participants (%) unless otherwise indicated. SIC: surgical informed consent, DiT: doctor-in-training

Variable	Proportion	Number
N=380	(%)	(n)
In elective cases, when is the best time/location to obtain SIC?		
Outpatient clinic	70	266
Ward	22.1	84
Holding bay	7.9	30
On operating table	0	0
Always checks competence when obtaining SIC		
Strongly agree	30.8	117
Agree	56.3	214
Neutral	9.5	36
Disagree	30	13
Strongly disagree	0	0
All elective surgical procedures should have valid SIC		
Strongly agree	58.9	224
Agree	38.4	146
Neutral	1.6	6
Disagree	1.1	4
Strongly disagree	0	0
All emergency surgical procedures should have valid SIC		
Strongly agree	23.9	91
Agree	39.2	149
Neutral	16.8	64
Disagree	20	76
Strongly disagree	0	0
Always use a structured proforma when obtaining SIC		
Strongly agree	21.8	83
Agree	41.8	159
Neutral	17.4	66
Disagree	17.1	65
Strongly disagree	1.8	7
Explains diagnosis and procedure indications when obtaining SIC		
Strongly agree	43.4	165
Agree	46.8	178
Neutral	7.4	28
Disagree	2.4	9
Strongly disagree	0	0
Understands risks/benefits/alternatives/complications when obtaining SIC		
Strongly agree	34.7	132
Agree	46.8	178
Neutral	9.5	36
Disagree	8.9	34
Strongly disagree	0	0
Often gets SICfor unfamiliar procedures		
Strongly agree	21.6	82
Agree	47.6	181
Neutral	4.2	16
Disagree	11.1	42
Strongly disagree	15.5	59
Often needs to obtain SICfor other specialities		
Strongly agree	31.6	120
Agree	35.3	134
Neutral	1.8	7
Disagree	16.8	64
Strongly disagree	14.5	55
Confident/comfortable obtaining SIC		
Yes	57.4	218
No	42.6	162

Univariate analysis

Univariate analysis showed significant positive associations between comfort/confidence with getting SIC and seniority (p<0.001), identification of SIC components (p<0.001), and prior training in SIC (p<0.001). Participants who had no formal training or had training in obtaining SIC during their internship were less comfortable/confident in obtaining SIC (p<0.001). There was no significant association between comfort and confidence in obtaining SIC and gender (p=0.581) (Table 4).

**Table 4:** Univariate analysis results with the likelihood of DiTs being comfortable/confident and SIC component identification as dependent variables Values are number of patients (%) unless otherwise indicated. *Pearson Chi-square analysis and Fisher’s exact test (for cell values <5), with significance at p<0.05 DiT: doctor-in-training, SIC: surgical informed consent, SMHS: South Metropolitan Health Services, EMHS: East Metropolitan Health Services, NMHS: North Metropolitan Health Services, PGY: postgraduate year

Variable (N=380)		Comfort/confidence	Comfort/confidence	p-value	Correct components	Correct components	p-value
		(n)	(%)		(n)	(%)	
Region	SMHS	63	46.3	<0.001*	107	78.7	0.002*
	NMHS	88	70.4		75	60	
	EMHS	67	56.3		74	62.2	
Gender	Male	99	60.4	0.581	94	57.3	0.001*
	Female	111	55		150	74.3	
	Undeclared	8	57.1		12	85.7	
Position	Intern	7	17.1	<0.001*	20	48.8	<0.001*
	Resident	101	52.9		108	56.5	
	Registrar	93	71		112	85.5	
	Fellow	17	100		16	94.1	
PGY	PGY 1 and 2	63	33.5	<0.001*	103	54.8	<0.001*
	PGY 3 and above	155	80.7		153	79.7	
Training level SIC	Medical school	110	68.8	<0.001*	125	78.1	<0.001*
	Internship	25	41		34	55.7	
	Residency	28	100		22	78.6	
	Registrar	5	100		5	100	
	No training	50	39.7		70	55.6	
Correct identification of SIC components	Yes	182	71.1	<0.001*	-	-	-
	No	36	29		-	-	
Consistent evaluation of competency	Yes	207	62.5	<0.001*	233	70.4	0.001*
	No	11	22.4		23	46.9	
Used information pamphlet	Yes	156	64.5	<0.001*	182	75.2	<0.001*
	No	62	44.9		74	53.6	
DiTs understood the risks/benefits/complications	Yes	218	70.3	<0.001*	256	73.8	<0.001*
	No	0	100		0	0	
DiTs had intimate knowledge of the Rogers versus Whitaker case	Yes	180	80.7	<0.001*	-	-	-
	No	38	24.2		-	-	
SIC policy awareness	Yes	169	60.8	0.026*	-	-	-
	No	49	48		-	-	
SIC policy familiarity	Yes	164	88.6	<0.001*	142	76.8	<0.001*
	No	54	27.7		114	58.5	
Consenting for unfamiliar procedures	Yes	113	43	<0.001*	152	57.8	<0.001*
	No	105	89.7		104	88.9	
Consenting for other specialties	Yes	114	44.9	<0.001*	159	62.6	0.005*
	No	104	82.5		97	77	
There is need for formal SIC training	Yes	207	56.1	0.003*	249	67.5	0.754
	No	11	100		7	63.6	
Preferred format of educational resource	Pocket guide/handbook	56	54.9	0.013*	65	63.7	0.005*
	Lecture series + handouts	31	77.5		36	90	
	Video/web-based modules	127	54.3		151	64.5	
	Other	4	100		4	100	

Furthermore, the univariate analysis also showed significant positive associations between the correct identification of SIC components and seniority (p<0.001), with more participants being able to identify these components correctly as their experience level increased. This was also true for gender (p<0.001), with females more likely to identify components correctly. The speciality of interest did not influence this. Participants who received formal SIC training were more likely to identify SIC components than those who did not (p<0.001). Of interest, participants who only underwent SIC training as interns demonstrated similar (poor) proportions of accurately identifying the components of a SIC (p<0.001) (Table 4).

Barriers and enablers

The critical enablers identified in the qualitative informants’ analysis included having previous training in SIC and working in well-supported departments with clear guidelines on SIC. The barriers highlighted were inexperience with SIC (especially when consenting for other specialities or unfamiliar procedures), time constraints, and the need for senior support (Table 5).

**Table 5:** Enablers and barriers to obtaining SIC for respondent DiT Values are the number of participants (%) unless otherwise indicated. SIC: surgical informed consent, DiT: doctor-in-training

Enablers	Barriers
Well-supported departments	Inexperience with SIC
Previous training in SIC	Time constraints
Clear guidelines on SIC	Lack of senior support

Thematic analysis

**Figure 1:**
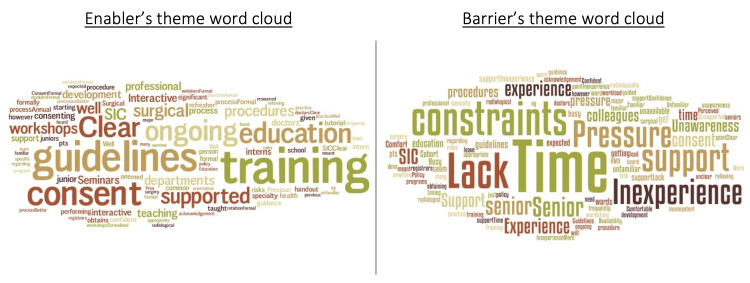
Thematic analysis cloud of enablers and barriers to obtaining SIC for respondent DiT SIC: surgical informed consent, DiT: doctor-in-training

A comprehensive logarithmic qualitative thematic analysis of free text item perspective responses was conducted. A pictorial representation of the key informants on enablers and barriers is demonstrated in Figure 1.

## Discussion

Obtaining an adequate SIC can be a complex and detailed process, the responsibility of which often befalls junior doctors in busy hospital settings, a homogenous observation between studies [10]. All clinicians have a moral and ethical obligation to engage fully in the SIC process, a conversation they ought to have with patients to ensure consistent delivery of individualized, safe, culturally sensitive, and appropriate healthcare. Poor SIC practices may expose the DiT to medicolegal risk, potentially causing patient harm. All stakeholders must actively engage in the SIC process to achieve appropriate shared decision-making.

This study shows that a significant proportion of SIC needs to be executed to the required legal standard (including disclosing relevant material risks), placing DiTs in a tenuous medicolegal predicament. It also reflects that DiTs may fall short of their duty of care when patients cannot fully participate in decision-making. This is further highlighted by the fact that while most DiTs who responded had SIC training in medical school, only a minority could adequately identify all the SIC components. Repeated SIC training may be more valuable when the DiTs are more experienced as they have better contextual understanding.

An alarming statistic showed that only half of the respondents felt confident and comfortable with the SIC process, further highlighting that we must do better in supporting DiTs. It demonstrates that DiTs may be acquiring consent despite feeling inadequate or experienced. Other studies have identified that DiTs may feel “pressured” to perform their job or meet expectations without “troubling” senior members, all to be considered competent [7]. The observed trend of improved confidence/comfort with SIC as doctors advance in seniority results from repeated exposure and experience, especially in a surgical-specific role. As part of the surgical team, DiTs’ experiences and learning increase exponentially through direct observation and practice, thus enhancing understanding and active engagement with the SIC process. During this period, as DiTs undergo this learning curve, they must be provided support, learning resources, and opportunities to solidify their experiences and knowledge of the SIC. By understanding the essential components of the SIC, DiTs can rely on their learned experiences and previous exposure when faced with unfamiliar or challenging situations, transferring these skills in situations like emergencies or where a patient is not competent.

“See one, do one, teach one” is a fundamental concept in Halsted’s model that surgeons alike are all too familiar with. Informal learning can be fostered by observing senior doctors perform SIC or, conversely, having senior doctors watch and critique junior doctors performing SIC. However, it is not a universally equal or reliable teaching method across hospital teams as it depends on time availability and the skillset of individual doctors. Formal training will improve SIC component familiarity and comfort/confidence in obtaining SIC. This could be done through professional skills workshops such as the Training in Professional Skills (TIPS) offered by the Royal Australasian College of Surgeons or hospital-based simulation skills workshops that allow for formal constructive feedback after observing DiTs performing these tasks. These opportunities should not be administrative-mandated learning modules as these tend to disengage learners [7]. There is a pressing need for a global initiative to standardize the education and training instruction of the SIC process as we move away from a paternalistic model to autonomous patient care, bearing in mind local cultural differences worldwide.

There are certain limitations to this study. Overall, the response rate for the survey was low at 23%. At the same time, low survey response rates are not uncommon, especially in surveys involving healthcare professionals [11-13]. This may reflect a level of response bias in our results. DiTs who felt strongly about SIC were likelier to participate, resulting in an over-representation of the cohort. An important observation from the study is that most respondents were comprised of junior doctors, i.e., interns and residents. Despite the low response rate, the study captured responses from the most susceptible group of junior doctors, early in their careers and often tasked with a realm of responsibilities, including obtaining consent.

To improve response rates, reminders at various periods could be sent out to prompt responses or provide small incentives for completing the surveys. Another option is to liaise with the institutions’ education officers to promote completing such surveys at the start of continuous professional development sessions. The study also included self-reported clinical practice, as assessing each documented consenting process for each respondent in the survey would be impractical. As such, the study would not be able to determine the competence of the DiT, with potential gaps between the DiTs’ confidence/comfort/skill with SIC and their basic skills in SIC.

Qualitative studies such as surveys are inherently prone to recall bias; for example, in this study, many DiTs reported not receiving formal training in obtaining SIC, which could be true. On the contrary, possibilities may include simply not remembering prior education in this area, particularly in cases of significant time lapse between education and undertaking the survey or where education received was considered inadequate. Lastly, this study did not analyze nor identify where respondents initially trained, with overseas trainees unlikely to be familiar with the nuances of the Australian medicolegal system compared to their domestic counterparts. In addition, different medical schools may vary in their approaches and emphasis on SIC training, leading to differences observed in the regions. This necessitates the pressing need for a global initiative to standardize the education and training instruction of the SIC process as we move from a paternalistic model to autonomous patient care.

## Conclusions

This study highlights the need for ongoing support and education for DiTs performing SIC. There is a greater need to emphasize SIC training, education, and practice beginning in medical school and then reinforced in clinical practice. Health institutions play an integral role in supporting DiTs in developing good SIC practices through regular training and providing access to clear guidelines. When clinicians and patients engage well in SIC, shared decision-making occurs, leading to individualized, safe, culturally sensitive, and appropriate healthcare delivery. A workforce well versed in the intricacies of the SIC process in tandem with a well-informed patient population will contribute to a sustainable healthcare system with consistent good health outcomes.

## Appendices

The survey questionnaire used in this study is shown in Table 6. 

**Table 6:** Supplemental online materials: pipetted survey questionnaire WA: Western Australia, QLD: Queensland

Survey items
Page 1
Project title: Perspectives on current attitudes, enablers, and barriers to obtaining surgical informed consent (SIC) for doctors-in-training
Q1. I hereby agree to be a participant in the above-named research.
Yes or No
Page 2: Demographics
Q2. Gender
0 = Male OR 1 = Female OR 2 = Undeclared
Q3. Postgraduate year
Enter number_____ yy in years
Q4. At what level of training did you receive formal instruction on obtaining surgical informed consent (SIC) for procedures?
0 = Medical school OR 1 = Internship OR 2 = Residency years OR 3 = Registrar years OR 4 = Fellowship years OR 5 = Never received formal training
Q5. At what age is a patient able to give a valid SIC?
Enter number_____ yy in years
Q6. Which of the following component/s are essential for obtaining a SIC?
0 = Evaluation of competence OR 1 = Patient education OR 2 = Recording of consent OR 3 = Patients’ signature OR 4 = Surgeons’ signature OR 5 = Date of consent OR 6 = Others - please specify
Q7. Are you aware of the nocebo effect of informed consent?
0 = No OR 1 = Yes
Page 3
Q8. Who do you think should obtain the SIC?
0 = The primary operator OR 1 = The consultant OR 2 = The fellow OR 3 = The trainee registrar OR 4 = The service registrar OR 5 = The resident OR 6 = The intern OR 7 = The medical student
Q9. In a situation where a patient is not capable of providing SIC, who is the next best person to provide a SIC? (Select all that apply.)
0 = The patient OR 1 = Next of kin OR 2 = Next of kin with enduring power of attorney OR 3 = Next of kin with enduring power of guardianship OR 4 = Family member OR 5 = Friend OR 6 = Hospital administrator
Q10. When do you think is the best time to obtain a SIC for a non-emergency procedure?
0 = The outpatient clinic OR 1 = On the ward OR 2 = Holding bay OR 3 = The anesthetic/induction room OR 4 = On the operating table
Page 4
Q11. How strongly do you Agree or Disagree with each of the following statements (5-point scale)?
1. Strongly agree, 2. Agree, 3. Neutral, 4. Disagree, 5. Strongly disagree
Valid SIC
a. I always check the patient’s competency to provide SIC.
b. All elective surgical procedures should have a valid SIC.
c. All emergency surgical procedures should have a valid SIC.
d. I use a structured proforma for obtaining SIC, e.g., WA or QLD Health Informed Consent Portal.
e. I always use the Royal Australia College of Surgeons’ procedure-specific consent.
Q12. How strongly do you Agree or Disagree with each of the following statements (5-point scale)?
1. Strongly agree, 2. Agree, 3. Neutral, 4. Disagree, 5. Strongly disagree
Components of SIC
a. When obtaining a SIC, I ensure that I explain the diagnosis and the indications for the procedure.
b. When obtaining a SIC, I ensure that I understand all the risks, benefits, complications, and alternatives to the procedure.
c. When obtaining a SIC, I ensure that my patient has understood the procedure.
d. I am familiar with the Rogers versus Whitaker case and the importance of material risk disclosure.
e. I am comfortable with the process of obtaining a SIC.
Q13. How strongly do you Agree or Disagree with each of the following statements (5-point scale)?
1. Strongly agree, 2. Agree, 3. Neutral, 4. Disagree, 5. Strongly disagree
Policies and procedures of SIC
a. I am aware of the presence of a hospital policy regarding informed consent for procedures.
b. I am familiar with the hospital policy regarding informed consent for procedures.
c. I often find myself in a situation where I have to obtain a SIC for a procedure I am not familiar with.
d. I often find myself having to obtain SIC for other specialties (e.g., percutaneous drainage done under radiology).
e. I believe that it is necessary to obtain formal training in obtaining surgical informed consent.
Page 5: Training
Q14. What is your preferred format for educational resources?
0 = Pocket guide/handbook OR 1 = Lecture series with handout OR 2 = Video or web-based learning module OR 3 = Others - please specify
Q15. What improvements would you like to see implemented in the training curricula?
Page 6
Q16. I would like to receive an executive summary of the research findings.
0 = Yes or 1 = No
Q17. If you answered yes to the above question, please provide your preferred email address for correspondence:
The contact details provided will be extracted separately from the study data to maintain anonymity.
Please note that this research group takes your confidentiality very seriously.
At no time will the raw data collected in this survey be released to any third party.
Thank you for taking the time to complete this survey. It is greatly appreciated!
End of the survey.
https://www.surveymonkey.com/r/SICWA
